# Mineralocorticoid Receptor Antagonism Prevents the Synergistic Effect of Metabolic Challenge and Chronic Kidney Disease on Renal Fibrosis and Inflammation in Mice

**DOI:** 10.3389/fphys.2022.859812

**Published:** 2022-04-07

**Authors:** Roberto Palacios-Ramirez, Ixchel Lima-Posada, Benjamin Bonnard, Marie Genty, Amaya Fernandez-Celis, Judith Hartleib-Geschwindner, Fabienne Foufelle, Natalia Lopez-Andres, Krister Bamberg, Frederic Jaisser

**Affiliations:** ^1^ Centre de Recherche des Cordeliers, Team Diabetes, Metabolic Diseases and Comorbidities, Sorbonne Université, Inserm, Université de Paris, Paris, France; ^2^ Cardiovascular Translational Research, Navarrabiomed (Miguel Servet Foundation), Instituto de Investigación Sanitaria de Navarra (IdiSNA), Pamplona, Spain; ^3^ Research and Early Development, Cardiovascular, Renal and Metabolism, Biopharmaceuticals R&D, AstraZeneca, Gothenburg, Sweden; ^4^ Université de Lorraine, INSERM Centre D’Investigations Cliniques-Plurithématique 1433, UMR 1116, CHRU de Nancy, French-Clinical Research Infrastructure Network (F-CRIN) INI-CRCT, Nancy, France

**Keywords:** CKD—chronic kidney disease, kidney, mineralocorticod receptors, inflammation, fibrosis

## Abstract

Obesity and/or metabolic diseases are frequently associated with chronic kidney disease and several factors associated with obesity may contribute to proteinuria and extracellular matrix production. Mineralocorticoid receptor antagonists have proven their clinical efficacy in diabetic kidney disease with preclinical data suggesting that they may also be efficient in non-diabetic chronic kidney disease associated to metabolic diseases. In the present study we developed a novel mouse model combining severe nephron reduction and High Fat Diet challenge that led to chronic kidney disease with metabolic alterations. We showed that the Mineralocorticoid Receptor antagonist canrenoate improved metabolic function, reduced albuminuria and prevented the synergistic effect of high fat diet on renal fibrosis and inflammation in chronic kidney disease mice.

## Introduction

Chronic kidney disease (CKD) is a progressive loss of kidney function over time and it is associated with high prevalence of cardiovascular morbidity and premature mortality. The prevalence of obesity, defined by a body mass index (BMI) above 30 kg/m2, and obesity-comorbidities also increases worldwide in men and women (Abrass, 2004). The kidney health has been associated to obesity: different renal alterations including micro- and macroalbuminuria, nephrotic-range proteinuria, glomerulomegaly with or without focal or segmental glomerulosclerosis, diabetic nephropathy, urolithiasis, renal carcinoma and diabetic nephropathy are more frequent in obese patients (Adelman, 2002; Praga, 2002; Wolf, 2003; Abrass, 2004; Rutkowski et al., 2006; Maric-Bilkan, 2013). The BMI is a predictor of end stage renal disease (ESRD) (Iseki et al., 2004). Several factors associated with obesity may contribute to proteinuria, glomerular hyperfiltration and extracellular matrix production including high protein and salt intake, hypertension, hyperinsulinaemia, hyperlipidaemia, increased sodium tubular reabsorption and synthesis of several adipose tissue-derived substances such as adipokines or plasminogen activator (Adelman, 2002; Rutkowski et al., 2006).

Our group showed that activation of the Mineralocorticoid Receptor (MR) has a deleterious effect on CKD progression (Barrera-Chimal et al., 2018, 2022). MR antagonists (MRAs) are promising therapeutic interventions in renal diseases (Barrera-Chimal et al., 2019) including diabetic kidney disease (DKD) (Barrera-Chimal and Jaisser, 2020). MR antagonism decreased albuminuria and renal fibrosis in preclinical studies in diabetic rats and mice associated or not with obesity (Cha et al., 2005; Han et al., 2006; Kosugi et al., 2010; Toyonaga et al., 2011; Bhuiyan et al., 2019; Dong et al., 2019; Wang et al., 2019) and clinical studies demonstrated MRA renal benefit in DKD patients (Lindhardt et al., 2016; Sun et al., 2017; Bakris et al., 2020; Wada et al., 2020; Filippatos et al., 2021).

The goal of the present study was to identify the underlying mechanisms by which MR antagonism limits CKD progression associated with metabolic disease. We developed a novel model combining 5/6 renal resection (Nx) and High Fat Diet (HFD) challenge in the C57Bl6 mouse strain known to be resistant to the development of renal fibrosis. We showed that pharmacological MR antagonism disrupts the deleterious crosstalk between metabolic challenge and renal organ damage thereby preventing renal inflammation and fibrosis.

## Materials and Methods

### Institutional Review Board Statement

All animal studies were conducted in accordance with the National Institutes of Health Guide and European Community directives for the Care and Use of Laboratory Animals (European Directive, 2010/63/UE). The animal studies were reviewed and approved by local animal ethics committee (agreement #22207-2019093015253955 v3).

### Animal Model

Animals were housed in the climate-controlled animal facility of the Cordeliers Research Center (INSERM U1136) in Paris (France) with a 12-h light/12-h dark cycle and provided free access to water and food (A04, Safediets; Augy, France). CKD was induced by 5/6 Nx in 8-weeks-old male mice (25–26 g). All surgeries were performed after subcutaneous ketamine/xylazine anesthesia (100 mg/kg) and placed on a heating pad to maintain core body temperature 37°C. Briefly, the left kidney was exposed and clamped, and the upper and lower poles were tied with a poly-glycolic acid suture line and absorbable haemostats (Surgicel, Johnson and Johnson Medical, Saint priest, France) were applied before clamp removal. The muscle and skin were then sutured, and the animals were returned to their cages. Sham surgery was carried out in anesthetized mice, left kidney was exposed for the same amount of time as nephrectomized mice without tying the poles. 7 days after recovery from the first surgery, the right kidney was removed. After the second surgery Time 0 started. 4 weeks after 5/6 Nx, renal function was measured by plasma creatinine and urea quantification with an automatic analyser (Konelab 20i; Thermo Fisher Scientific, Vernon Hills, IL, United States) to ensure all CKD animals were homogeneous with increased levels compared to sham mice. Mice were then randomly split in five groups: Sham (*n* = 7), Sham with 60% fat diet (Research Diets^®^; New Brunswick, United States) (Sham HFD) (*n* = 7), CKD (*n* = 9), CKD with 60% fat diet (Research Diets^®^) (CKD-HFD) (*n* = 9) and CKD-HFD treated by subcutaneous injection with 30 mg/kg/day canrenoate (Sigma-Aldrich^®^; Saint-Louis, Missouri, United States) in saline solution (CKD-HFD-CAN) (*n* = 9). Sham and CKD HFD animals received daily saline solution injection. Duration of HFD and canrenoate treatment was 8 weeks.

Animals were euthanized 12 weeks after CKD or Sham surgery. Kidney and epididymal visceral adipose tissue (EVAT) were collected and rinsed in ice-cold Dulbecco’s phosphate buffered saline (DPBS; ThermoFisher; Waltham, Massachusetts, United States). The kidney was cut in two parts, one for histology and the other one was snap frozen in liquid nitrogen and stored at −80°C for molecular analysis. EVAT was weighed and snap frozen in liquid nitrogen and stored at −80°C.

### Glucose and Insulin Tolerance Tests

At 6–7 weeks after surgery, a glucose tolerance test (GTT) and insulin tolerance test (ITT) were performed as described (Nguyen et al., 2019). For the GTT, mice were fasted for 5 h and then received an intraperitoneal injection of a 50% w/v glucose (Sigma-Aldrich^®^) solution at a dose of 2 g/kg body weight. Blood was sampled via tail tip bleed and blood glucose concentrations were measured using a glucometer (Accu-Check Performa^®^, Roche, Basel, Switzerland) prior to glucose administration (T0), and at (T) 15, 30, 60, 90- and 120-min post-bolus. For the ITT, mice were fasted for 2 h and then received an insulin (Humalog^®^, VIDAL, France) intraperitoneal injection of 0.5 U kg^−1^ of body weight. Tail tip bleeds were collected to measure blood glucose levels using a glucometer as described above, at (T) 0, 15, 30, 60- and 90-min post-injection.

### Biochemical Studies

24-h urine were collected using metabolic cages in all studied groups. Albuminuria was determined by Mouse Albumin ELISA Kit (Crystal Chem; Elk Grove Village, IL, United States), 8 weeks after CKD or Sham surgery. Glycated hemoglobin (HbA1c) was measured using a specific kit (A1CNOW/Detecteur hemoglobine glyquee; Frances Neir, Faches-Thumesnil, France) in blood sampled via tail tip bleed. Plasma K+ and hematocrit were measured in blood collected via tail tip bleed and analyzed with (epoc^®^ Blood Analysis System, Siemens Healthcare GmbH, Erlangen, Germany).

### Magnetic Resonance Imaging

The whole-body fat content was determined by MRI in all groups at 8 weeks after surgeries (Bruker BioSpin, Billerica, MA).

### Histology

Kidneys were collected and a section was immersed in paraformaldehyde fixative solution for 24 h (Sigma-Aldrich^®^). After fixation, the sections were dehydrated and embedded in paraffin.

Histological determinations were performed in 5 μm-thick paraffin-embedded serial sections following the protocol of Leica BOND-Polymer Refine Detection automatic immunostainer (Leica). All solutions were filled into the bottle-Bond Open Container (Leica, Wetzlar, Germany) and registered on computer using the Leica Biosystem program. The immunostaining program protocol include: Fixative solution, Bond wash solution, blocking with common immunohistochemistry 5% milk and incubated with the primary antibody for CD3 (Santa Cruz Biotechnology, Heidelberg, Germany), CD4 (Santa Cruz Biotechnology), CD45 (Santa Cruz Biotechnology) and CD68 (Abcam, Cambridge, United Kingdom). After primary antibody incubation, slides were incubated with secondary poly-HRP-IgG. The signal was revealed by using DAB substrate. Incubation with no primary antibody was carried out in negative controls.

For collagen quantification, tissues were hydrated in water and slides were incubated with 1% Sirius red in picric acid for 30 min. Histological and immunohistochemistry preparations were imaged using bright field or polarized light in an automated image analysis system, as appropriate (Nikon, Champigny sur Marne, France). Digital image analyses were additionally performed for a further histoanatomical characterization. In brief, arbitrary fields per section were imaged at 50 or ×400 magnification, as appropriate. The content of thin and thick collagen fibers was quantified using ImageJ software by performing a binary conversion of the images on red channel to quantify the positive % of area occupied by these targets. All quantifications were normalized to the area of the tissue analyzed. All the experiments were performed by two different blinded observers. In the figures the most representative images were shown.

### Gene Expression Analysis by Real-Time Reverse Transcription PCR

Frozen tissues (kidney and EVAT) were homogenized in TRIzol (Life Technologies, Illinois, United States) using FastPrep beads (MP-Bio, CA, United States). RNA was extracted following the protocol of the manufacturer. Up to 2 ug of RNA were mixed with 200 U of Moloney murine leukemia virus reverse transcriptase (M-MLV, Invitrogen, NY, United States), random primers and dNTP’s (Invitrogen) to generate cDNAs. qPCR was performed as described (Bonnard et al., 2021). Briefly, transcript levels were analysed in a CFX396 apparatus (Biorad, CA, United States). The reactions were prepared in duplicate for each sample using the IQ SYBR Green supermix Kit (Biorad). To normalize gene expression, we used HPRT as an endogenous control. The relative quantification of each gene expression was calculated with the comparative threshold cycle (2^−ΔΔCt^) method. The sequences of the specific primers are detailed in Supplementary Table S1.

### Statistical Analysis

The hypotheses we wanted to test in this study were 1) does HFD challenge increase renal injury in a CKD model with 5/6 nephrectomy done in C57B6 mouse strain, 2) if yes, does an MRA limit the renal damages. So we designed our experimental protocol to assess these points with the limitation that some groups of interest, especially for statistical analyses, are missing. Adherence to 3R animal welfare principles, considering the demanding surgical procedure required to generate CKD mice with 5/6 nephrectomy, underscore the difficulty of having several groups in parallel with sufficient number of animals per group. Hence we judge a 1-way ANOVA analysis between the tested groups is an appropriate method to assess statistical significance to test our hypotheses.

The results are presented as the mean ± SE. The differences between the groups were assessed by one-way ANOVA using the Tukey post-test and two-way ANOVA with the Sidak post-test for the insulin and glucose tolerance tests. Differences in the means between two groups for non-repeated variables were compared by Student’s t test. Analyses were performed using GraphPad Prism 7.4. Results were considered significant when the *p* value was <0.05.

## Results

**FIGURE 1:**
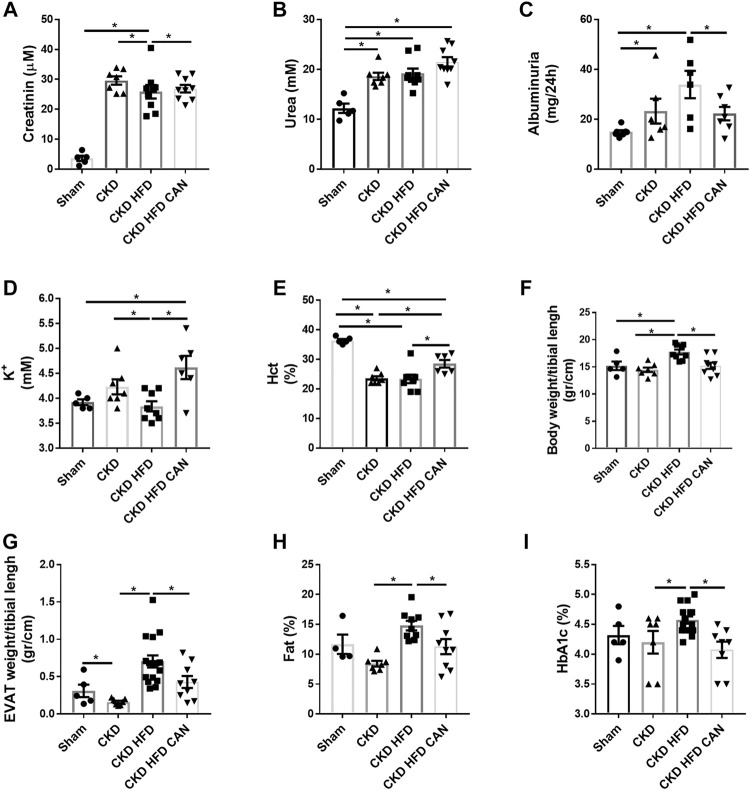
Physiological parameters of the groups Sham, CKD, CKD HFD and CKD HFD CAN. **(A)** Plasma creatinine; **(B)** plasma urea; **(C)** Albuminuria; **(D)** plasma potassium; **(E)** hematocrit (Hct); **(F)** body fat percentage; **(G)** body weight; **(H)** epididymal adipose tissue (EVAT) weight and **(I)** glycated hemoglobin (HbAc1). Statistical analysis by one-way ANOVA with Tukey post-test, *p < 0.05 *n* = 5–9.

At sacrifice, the mice undergoing 5/6 nephrectomy (CKD mice) showed higher levels of creatinine and urea plasma levels compared to the Sham mice with no differences between the CKD and the CKD HFD mice (Figures 1A,B). The CKD and the CKD HFD mice presented higher albuminuria than Sham (Figure 1C). The plasma potassium levels were unchanged in the CKD mice compared to the Sham mice, while they were lower in the CKD HFD mice compared with the CKD (Figure 1D). The hematocrit was lower in both the CKD and the CKD HFD mice compared to the Sham group (Figure 1E).

**FIGURE 2:**
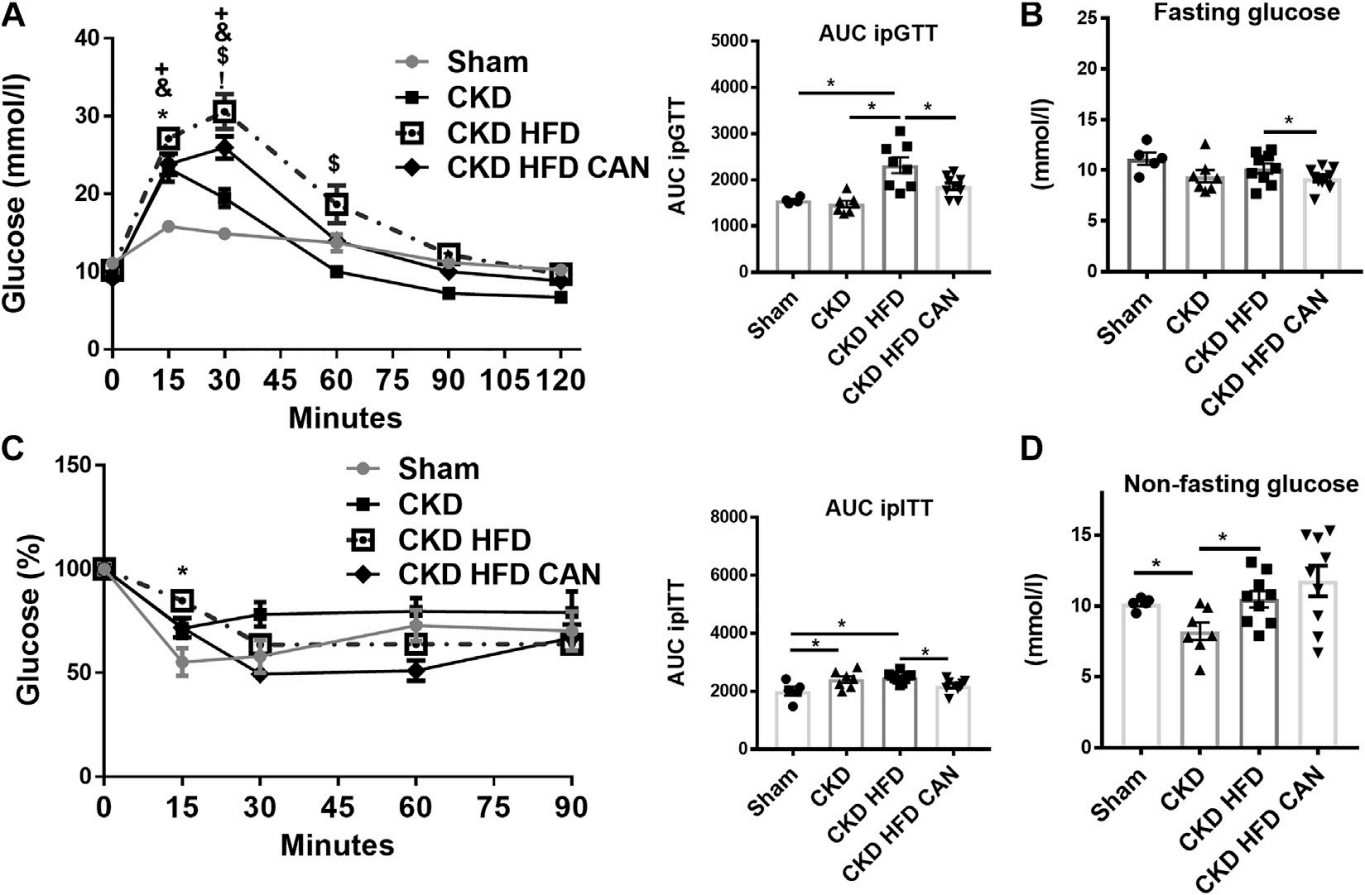
Characterization of the metabolic impact and effect of the MRA canrenoate. Mice were subjected to a glucose tolerance test (GTT) and an insulin sensitivity test (ITT). **(A)** Glucose tolerance test and area under the curve **(B)** fasting glucose **(C)** Insulin tolerance test represented as a decrease of the percentage of basal glucose over time, and area under the curve, **(D)** non-fasting glucose. Data represent the mean ± SEM. Statistical analysis by two-way ANOVA with Sidak post-test, *p < 0.05 CKD HFD vs. Sham, ^#^ *p* < 0.05 CKD HFD vs. CKD, +*p* < 0.05 CKD HFD CAN vs. CKD HFD, ^&^ *p* < 0.05 CKD groups vs. Sham; and one-way ANOVA with Tukey post-test *p < 0.05, Sham vs. CKD; ^+^ *p* < 0.05, Sham vs. CKD HFD; ^&^ *p* < 0.05, Sham vs. CKD HFD CAN; ^$^ *p* < 0.05, CKD vs. CKD HFD *n* = 5–9; ^!^ *p* < 0.05, CKD vs. CKD HFD CAN or one-way ANOVA; with Tukey post-test *p < 0.05 *n* = 5–9 for ITT and GTT AUC and panels B and C.

The CKD mice fed with HFD had increased body weight and EVAT size compared to both the Sham and the CKD mice (Figures 1F,G). The CKD mice had reduced fat mass compared to the Sham and the CKD HFD mice (Figure 1G). They also had higher levels of fat content and glycated hemoglobin (HbA1c) than the CKD mice (Figures 1H,I). The CKD HFD mice showed impaired glucose tolerance compared to both the Sham and the CKD mice (Figure 2A). This can be observed in the area under the curve (AUC) plot (Figure 2A). The fasting glucose was similar between the Sham, the CKD and the CKD HFD mice (Figure 2B). The insulin response was impaired in the CKD HFD compared to the Sham mice (Figure 2C). The AUC of the insulin tolerance test was similar between the CKD and the CKD HFD mice and higher than in the Sham (Figure 2C). The non-fasting glucose was lower in the CKD mice compared to the Sham and normalized in the CKD HFD (Figure 2D). HFD alone impaired glucose tolerance (Supplementary Figure S1A). Both, fasting and non-fasting plasma glucose levels were increased in the HFD mice compared to the Sham (Supplementary Figure S1B–D). The response to insulin was not different (Supplementary Figure S1C).

**FIGURE 3:**
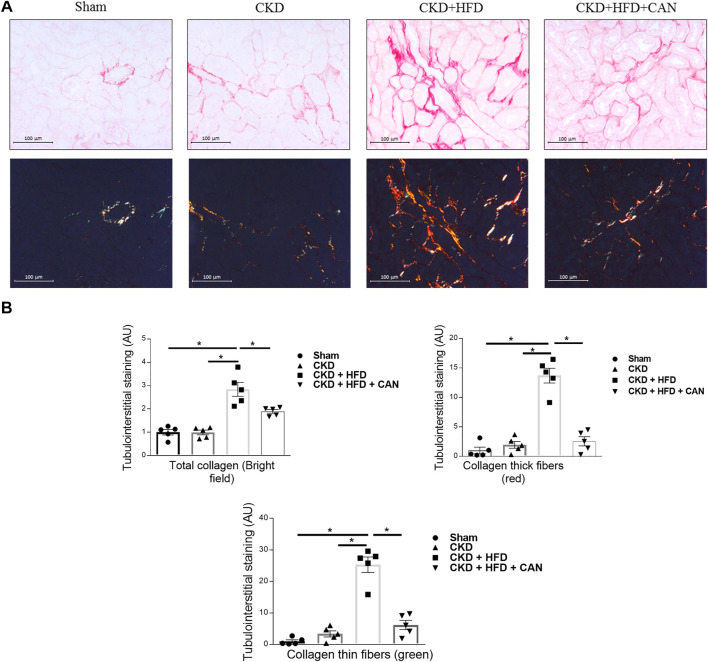
Characterization of the renal injury and effect of the MRA canrenoate. **(A)** Sirius red staining and collagen fiber content (bar scale 100 μm) showing the fibrosis in the kidney of Sham, CKD, CKD HFD and CKD HFD CAN mice and **(B)** its quantification. Data represent the mean ± SEM. Statistical analysis by one-way ANOVA; with Tukey post-test *p < 0.05 *n* = 5–9.

**FIGURE 4:**
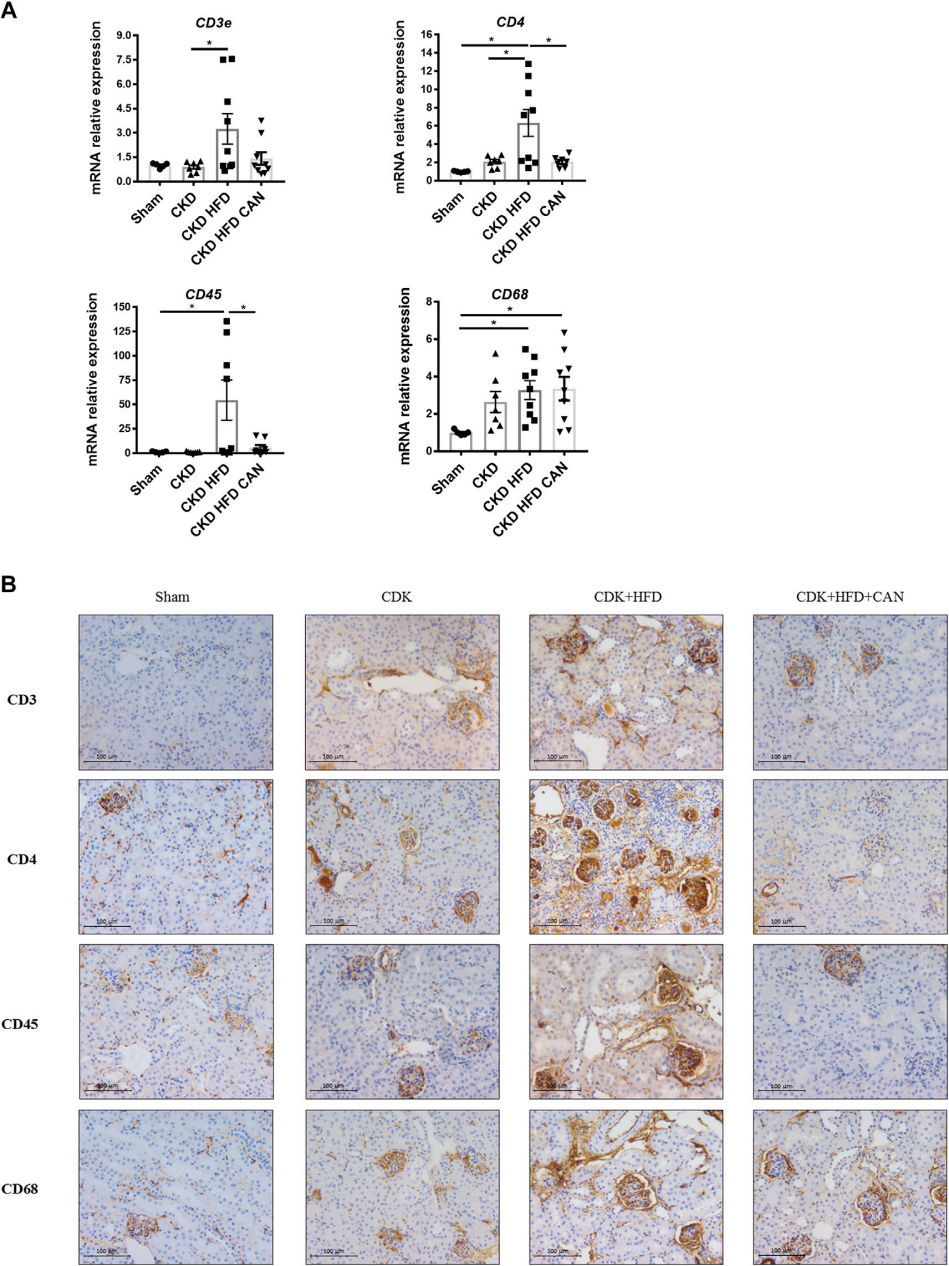
Renal immune cell infiltration and effect of the MRA canrenoate. **(A)** mRNA levels of *CD3, CD4, CD45 and CD68* **(B)**. Immunostaining of CD3, CD4, CD45 and CD68. Data represent the mean ± SEM. Statistical analysis by one-way ANOVA; with Tukey post-test, *p < 0.05 *n* = 5–9.

As shown in sirius red staining, renal interstitial fibrosis was not altered in the CKD mice on a C57Bl6 mouse background (Figures 3A,B). However, renal interstitial fibrosis is increased in the CKD HFD mice compared to the Sham and the CKD alone (Figure 3B). Interestingly, both thicker and thinner collagen fibers were increased in the CKD mice fed a HFD (13.7- and 25.3-fold respectively, *p* < 0.0001). Tissue infiltration of immune cells has an important role in the fibrosis observed in both acute and chronic renal damage (Panizo et al., 2021). We found increased levels of mRNA levels of CD45, CD68 and CD4 in the kidneys of the CKD HFD mice compared to the Sham and of CD3e and CD4 compared to the CKD alone (Figure 4A). This effect was confirmed by immunostaining with no differences between the Sham and the CKD mice and an increased staining for CD3, CD4, CD45 and CD68 in CKD HFD compared to both the Sham and the CKD (Figure 4B). Of note HFD alone did not impair the renal function (Supplementary Table S2), renal fibrosis (Supplementary Figure S2) or inflammation (Supplementary Figure S3). Taken together, these results suggest that HFD challenge together with 5/6 nephrectomy in C57Bl6 mice leads to a combined metabolic/renal damage more severe than 5/6 nephrectomy alone.

MRA treatment can result in improved metabolic features (Marzolla et al., 2020). We therefore analysed whether treatment with canrenoate over 8 weeks affected metabolic parameters in the context of CKD. The treatment with canrenoate reduced body weight, fat mass, EVAT size and HbA1c (Figures 1F–I). The impaired glucose tolerance induced by the HFD challenge in CKD mice (CKD HFD group) was alleviated in the canrenoate treated group (Figure 2A). Insulin response was also improved as shown by the AUC plot (Figure 2C). The fasting glucose was lower in the canrenoate-treated CKD HFD mice (Figure 2B) while the non-fasting glucose was similar in both groups (Figure 2D).

Canrenoate treatment of CKD HFD mice for 8 weeks did not improve plasma creatinine or urea levels (Figures 1A,B). However, MRA treated animals had lower albuminuria and increased hematocrit compared to vehicle treated animals (Figures 1C,E). Canrenoate treatment also resulted in elevated plasma potassium levels in the CKD HFD CAN group compared to CKD HFD group (Figure 1D). Interstitial fibrosis was lower in the CKD HFD CAN group compared to the CKD HFD alone (Figures 3A,B). The MRA treatment decreased the mRNA levels of *CD3E*, *CD4* and *CD45* compared to the CKD HFD (Figure 4A), while gene expression of the *CD68* marker is unaffected (Figure 4A). The immunostaining supported these results with clear reduction of CD3, CD4 and CD45 staining with only a mild decrease on CD68 staining (Figure 4B) indicating that the MRA canrenoate prevented renal inflammation induced by combined nephron reduction and metabolic challenge.

## Discussion

In the present study we characterized the metabolic alterations and the kidney damage in a new kidney disease model combining nephron reduction (5/6 nephrectomy) and HFD in mice on a C57Bl6 genetic background. We showed that targeting the MR pathway using the MRA canrenoate improved metabolic parameters and decreased albuminuria and renal interstitial fibrosis and inflammation.

Several mouse models of metabolic kidney disease have been developed (Heinz-Taheny et al., 2018). The mouse models reproducing advanced features of the disease are based on genetic models such as ob/ob mice in the BTBR background or db/db along with unilateral nephrectomy, high protein diet or AngII-induced hypertension (Sharma et al., 2003; Hartono et al., 2014; Nørgaard et al., 2019). However, it remains difficult to mimic diabetic nephropathy in the common C57Bl6J background, classically used for mechanistic studies based on gene inactivation, but which is very resistant to kidney damage (Rabe and Schaefer, 2016). The 5/6 nephrectomy is a well-established model of CKD triggering an inflammatory response and glomerular hyperfiltration resulting in glomerulosclerosis and tubulointerstitial damages, leading to chronic kidney disease (Bao et al., 2018). Nevertheless, the C57Bl6 background is considered a resistant strain to this CKD model (Ma and Fogo, 2003) as fibrosis for example barely develops. Since most of the cell-specific gene inactivation models are done on this C57Bl6 background, this combined model of 5/6 nephrectomy with HFD will be particularly useful to decipher the cell-specific role of MR in this context, using endothelial, smooth muscle or myeloid MR KO mouse models. In particular, we believe that the addition of HFD is improving the translation of preclinical to clinical studies as it recapitulates several features of CKD in patients with metabolic disease. Obesity is closely associated with renal damage (Adelman, 2002; Praga, 2002; Wolf, 2003; Abrass, 2004; Rutkowski et al., 2006; Maric-Bilkan, 2013) and it has been reported that a high fat diet causes lipid accumulation in the proximal tubule, increases the inflammation which damages renal structure and function (Chen et al., 2021). Therefore renal inflammation might be expected in mice fed a HFD. However, it has also been reported that HFD alone is not a good method to cause damage in the mouse kidney, at least in C57BL6/J, (Wicks et al., 2016). Indeed, HFD alone is not reported as an appropriate method to generate a robust model of metabolic kidney injury in mice (Heinz-Taheny et al., 2018). Therefore, we used a combined model with two different challenges to try to recapitulate the CKD progression associated with metabolic challenge.

The involvement of visceral adipose tissue (VAT) in kidney damage associated with obesity is well established. The VAT is metabolically active and produces inflammatory adipokines. This cytokine production is increased in obesity and it is known to play a role in the development and progression of kidney disease (Whaley-Connell and Sowers, 2017).

Another important point is the regulation of the renin angiotensin aldosterone system (RAAS) by excess VAT and circulating insulin in obesity leading to an inappropriate activation of tissue and systemic RAAS in obesity (Whaley-Connell and Sowers, 2017; Packer, 2018). Moreover, VAT mitochondrial function is regulated by MR and affects vascular function of mesenteric arteries (Lefranc et al., 2019). In one clinical trial testing eplerenone in heart failure (EMPHASIS-HF), waist circumference was a major determinant of the response to treatment (Packer, 2018): the reduction of the risk of cardiovascular death and heart failure hospitalization was more than two times greater in patients with abdominal obesity than in those with a normal waist circumference (Packer, 2018). Therefore, the use of MR antagonism could be particularly appropriate in patients with VAT accumulation such as those with obesity or metabolic syndrome. We therefore attempted to develop a metabolic-associated kidney disease model in WT C57BL/6J mice by combining nephron reduction and metabolic challenge, mimicking what is present in up to 30% of the ESRD patients or patients with stage 3 diabetic nephropathy (Reutens, 2013). Renal function was impaired in CKD HFD mice with increased albuminuria suggesting altered glomerular function. Importantly interstitial fibrosis was increased in this model compared to mice with nephron reduction alone. As reported in rats by Hosoya et al. (Hosoya et al., 2015), severe nephron reduction alone induced insulin resistance and reduced fasting glucose. However, glucose tolerance, fasting glucose or HbA1c were not affected in the C57Bl6 CKD mice. CKD mice fed HFD showed increased body weight, body fat mass percentage, EVAT size, HbA1c, impaired glucose tolerance and insulin resistance, all features present in other models such as db/db or ob/ob mice (Giralt-López et al., 2020). Of note HFD alone has no significant renal impact despite even worse metabolic impairment compared to CKD HFD mice, suggesting that the combination of nephron reduction and metabolic challenge has a synergistic deleterious effect.

MR is expressed in myeloid cells including macrophages (Usher et al., 2010). Macrophages isolated from DOC/salt mice showed a proinflammatory expression profile with increased expression of interferon gamma, tumor necrosis factor alpha (TNFα), matrix metalloproteinase 12 and other molecules. This proinflammatory effect was reduced in macrophages isolated from DOC/salt MR null myeloid cells (Shen et al., 2016). Regarding the impact of MR expressed in macrophages on organ damage, it has been reported that myeloid MR-deficient mice were protected against anti-GBM glomerulonephritis, mimicking the benefit of eplerenone, suggesting that macrophages are a key component of the MR-induced kidney damage (Tesch and Young, 2017). In a model of bilateral kidney ischemia/reperfusion injury in mice, the treatment with the MRA finerenone protected against chronic dysfunction and fibrosis, an effect that was mimicked by myeloid MR deletion (Barrera-Chimal et al., 2018). Altogether, these studies support an important role of myeloid MR-activation in end organ damage.

We found high levels of leukocyte markers supporting an increase of the immune cell infiltration. The increase in the T cell infiltration is related with the fibrosis development in different models of kidney damage (Hartono et al., 2014; Liu et al., 2015; Liang et al., 2018; An et al., 2020). We found increased levels of CD3, a T-cell surface glycoprotein, in the kidney of the CKD HFD mice together with higher renal fibrosis compared to Sham and CKD alone. We also observed an increased staining of CD4 in the kidney of the CKD HFD compared to the other groups. Infiltration of CD4 positive T-cells subpopulation is also associated with kidney fibrosis (Tapmeier et al., 2010; Panizo et al., 2021). In addition, it has been shown that CD45^+^ and CD68^+^ immune cells may promote inflammation in the kidney, thereby perpetuating kidney fibrosis (Sakai et al., 2006; Kisseleva and Brenner, 2008; Wada et al., 2011; Liu et al., 2015; Qiu et al., 2019); the expression of these markers was strongly increased in the kidney tissue of CKD HFD mice.

The impact of the MRA canrenoate on the glucose tolerance along with the limitation of body weight gain, the decrease of the glycated Hb and the lower fasting glucose indicates an improved metabolic status. The metabolic benefit of MRA spironolactone on glucose tolerance was reported in 5/6 nephrectomised rats (Hosoya et al., 2015) and in mice fed on 60% HFD (Marzolla et al., 2020) but not in a composite model that combines impaired renal function and metabolic alterations. Whether metabolic improvement also occurred in CKD patients with type 2 diabetes and/or obesity treated with an MRA and participate to the therapeutic benefit remains to be explored.

Preclinical studies consistently reported improved renal function with decreased albuminuria and renal fibrosis, preservation of glomerular structure and reduced podocyte lesions (Cha et al., 2005; Han et al., 2006; Kosugi et al., 2010; Toyonaga et al., 2011; Bhuiyan et al., 2019; Dong et al., 2019; Wang et al., 2019). Several clinical studies reported the benefit of MR antagonists in diabetic kidney disease outcomes (Lindhardt et al., 2016; Sun et al., 2017; Wada et al., 2020) but clinical impact on non-diabetic CKD remains to be demonstrated. Hou et al. reported in a meta-analysis that adding spironolactone to standard treatment in patients with DKD could prevent or slow DKD progression with a reduction of proteinuria (Hou et al., 2015). The addition of the non-steroidal MR antagonist finerenone on standard care in DKD patients decreased albuminuria and UACR (Katayama et al., 2017; Bakris et al., 2020) and led to improvements in renal endpoints in the FIDELIO-DKD trial (Bakris et al., 2020). In the present preclinical study, the MRA canrenoate had no effect on plasma creatinine or urea levels. This was also reported in several rodent models of CKD (Michea et al., 2008; Nemeth et al., 2009; Bonnard et al., 2018). We noticed an improvement of hematocrit by the MRA treatment which may indicate improved renal health. Importantly less interstitial fibrosis was also observed upon MRA treatment of CKD HFD mice. Similar results have been described in a diabetic nephropathy mouse model treated with the MRA eplerenone (Zhou et al., 2016), in high salt-treated mice with genetic type 2 diabetes treated with the non-steroidal MRA exaserenone (Bhuiyan et al., 2019) and in spontaneously type 2 diabetic rats treated with spironolactone (Cha et al., 2005).

MRA reduced immune cell infiltration as reported in different animal models: eplerenone reduced the infiltration of T-cells in a model of glomerulonephritis (Zitt et al., 2011) and spironolactone prevented macrophage infiltration in rat models of type 1 (Fujisawa et al., 2004) or type 2 diabetes (Han et al., 2006). The administration of eplerenone also suppressed macrophage infiltration in obese ob/ob and db/db mice (Hirata et al., 2009) In the combined CKD-HFD model we analyzed in the present study, the MRA canrenoate reduced the staining of macrophages and T cells in the kidney, indicating an important anti-inflammatory effect that could participate to the improved renal outcomes we observed.

In this study, the effect of different treatment paradigms on the gut microbiota was not analysed. The fiber intake was not significantly different among groups, however the HFD and CKD are known to be associated with dysbiosis (Murphy et al., 2015; Plata et al., 2019). We cannot exclude a role of microbiota dysbiosis in the effects reported in this study.

In conclusion we characterized a novel non-genetic mouse model of kidney disease associated to metabolic impairment on a C57Bl6 genetic background. This may be a great advantage for future mechanistic studies using cell-specific gene inactivation. We showed that the MRA canrenoate has a beneficial impact on both metabolic and renal features as well as renal fibrosis and inflammation in this model.

## Data Availability

The raw data supporting the conclusion of this article will be made available by the authors, without undue reservation.

## Abbreviations

CKD, chronic kidney disease; ESRD, end stage renal disease; MR: mineralocorticoid receptor; MRAs, MR antagonists; HFD, high fat diet; Nx, renal resection; EVAT, epididymal visceral adipose tissue; GTT, glucose tolerance test; ITT, insulin tolerance test; HbA1c, glycated haemoglobin; AUC, area under the curve.
