# Supplementary material for: Mineralocorticoid Receptor Antagonism Prevents the Synergistic Effect of Metabolic Challenge and Chronic Kidney Disease on Renal Fibrosis and Inflammation in Mice
Source: Front Physiol. 2022 Apr 7;13:859812. doi: 10.3389/fphys.2022.859812 (PMC9022039; doi:10.3389/fphys.2022.859812)
Supplement: Supplementary file 1 [file DataSheet2.PDF]

Supp Figure 1

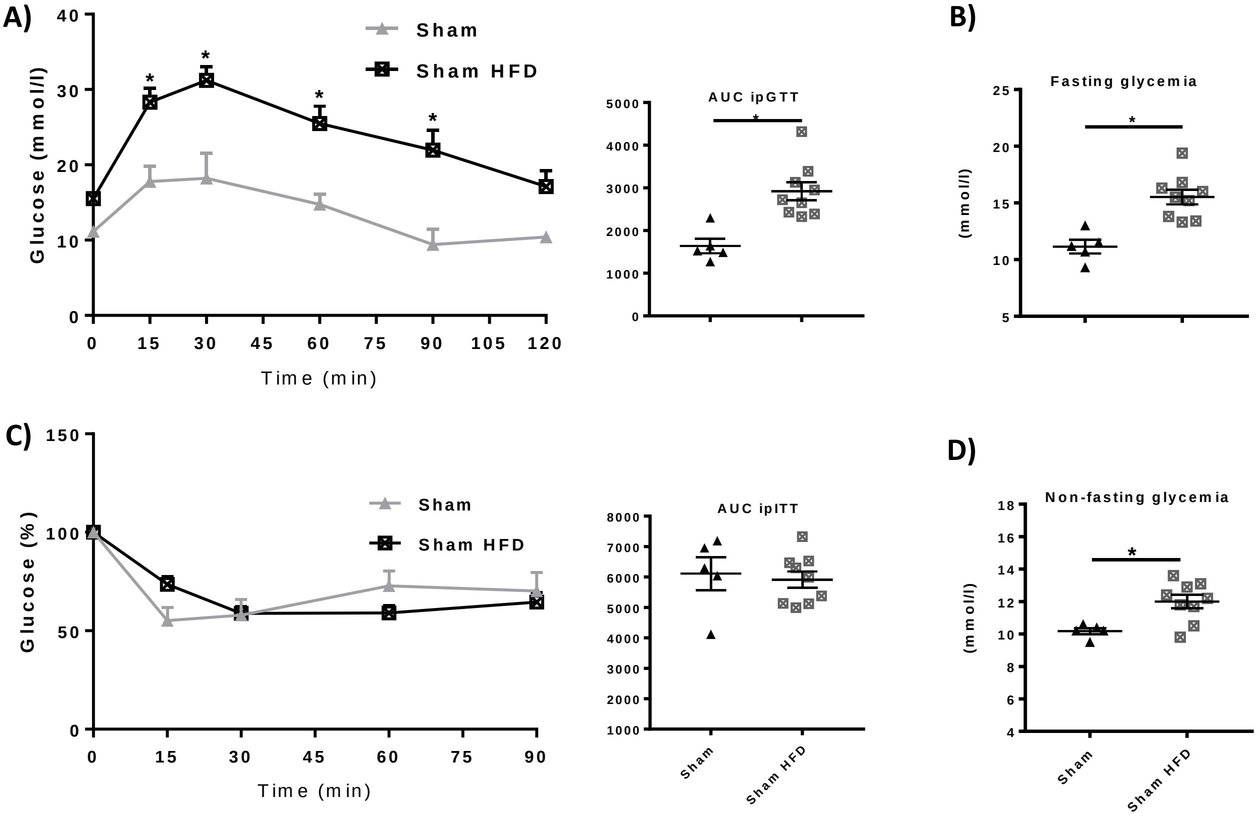

Supplemental Figure 1. Characterization of the metabolic impact of HFD in sham mice.

(A) Glucose tolerance test and area under the curve (B) fasting glucose (C) Insulin tolerance test represented as a decrease of the percentage of basal glucose over time and area under the curve, (D) non-fasting glucose. Data represent the mean ± SEM. Statistical analysis by two-way ANOVA with sidak post test, *P<0.05, and student t-test; *P<0.05 n=5-9.

Supp Figure 2

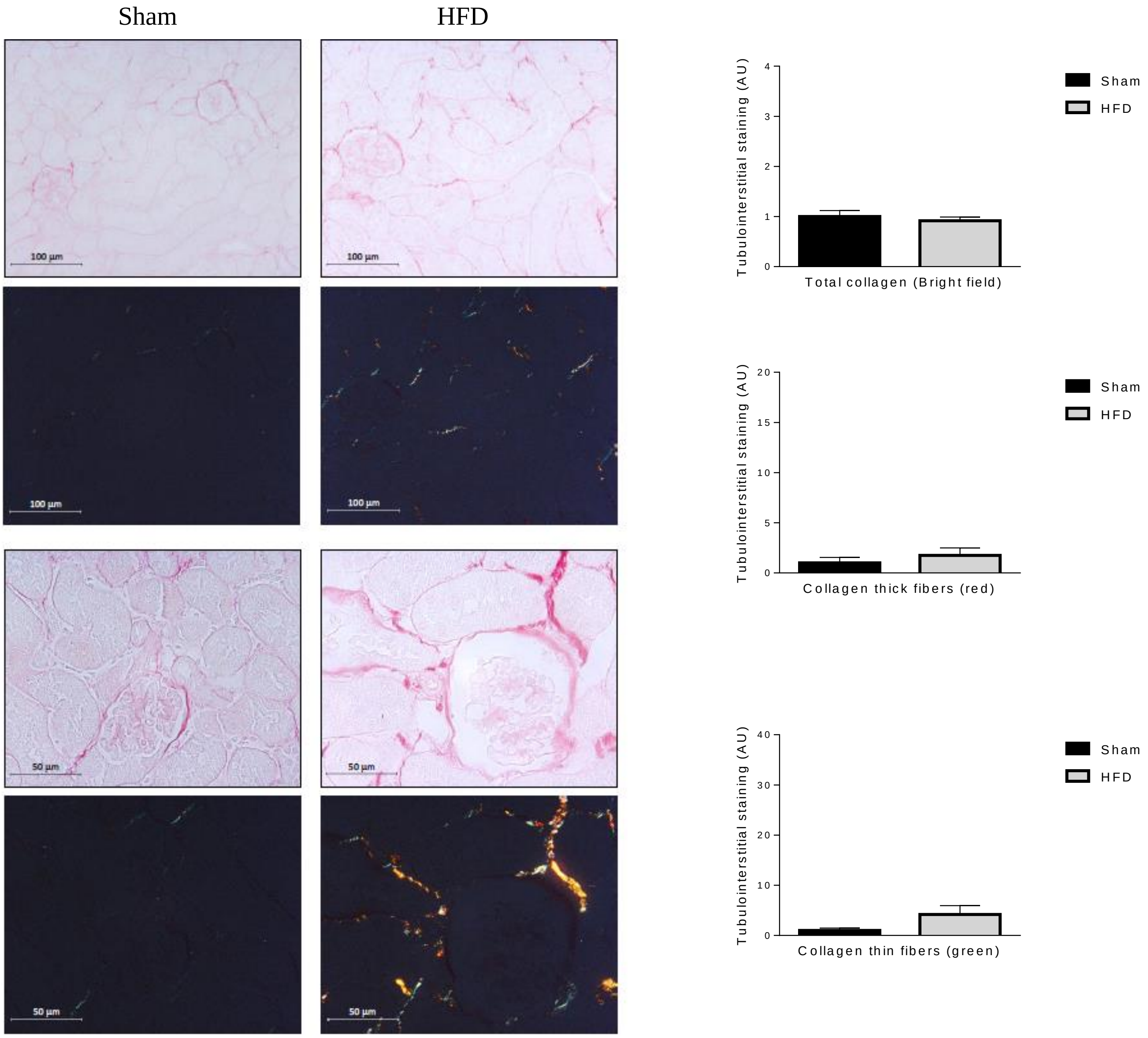

Supplemental Figure 2. Characterization of the profibrotic impact of HFD. Sirius red stain micrography showing the fibrosis in the kidney of Sham and Sham HFD mice and siriues red stain micrography carried out under polarized light showing the different types of fibers and its quantification. Data represent the mean ± SEM. Statistical analysis by student t-test; *P<0.05 n=5-9.

Supp Figure 3

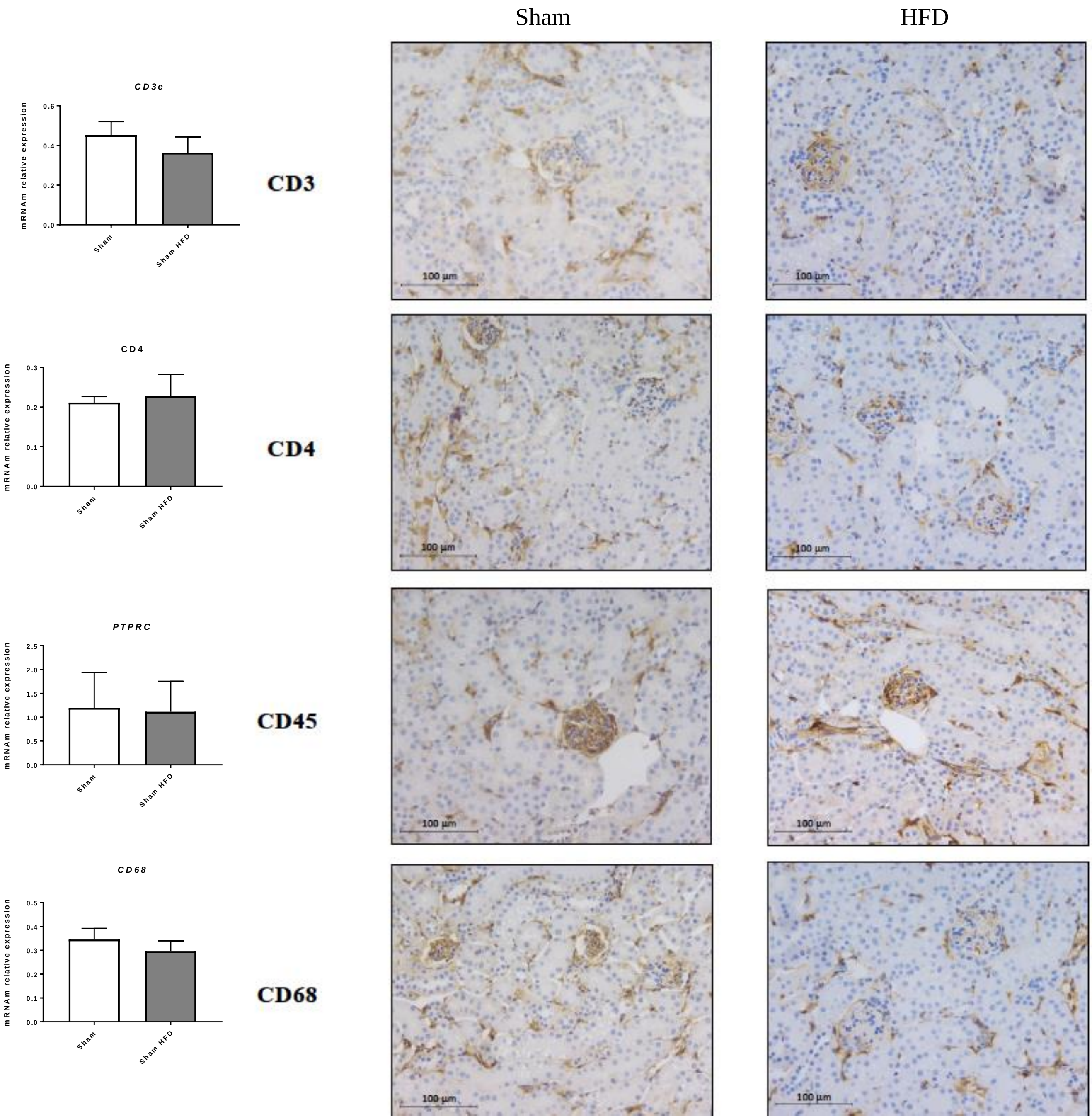

Supplemental Figure 3. In the Left, mRNA levels of *CD3*, *CD4*, *PTPRC* and *CD68*. In the right, immunostaining micrographies showing the infiltration of CD3$^+$, CD4$^+$, CD45$^+$ and CD68$^+$ cells. Data represent the mean ± SEM. Statistical analysis by student t-test; *P<0.05 n=5-9.
