# Supplementary material for: Mineralocorticoid Receptor Antagonism Prevents the Synergistic Effect of Metabolic Challenge and Chronic Kidney Disease on Renal Fibrosis and Inflammation in Mice
Source: Front Physiol. 2022 Apr 7;13:859812. doi: 10.3389/fphys.2022.859812 (PMC9022039; doi:10.3389/fphys.2022.859812)
Supplement: Supplementary file 2 [file DataSheet1.PDF]

Sup table 1: List of primers

Genes	Forward	Reverse
**HPRT**	CTCAACTTTAACTGGAAAGAATGTC	TCCTTTTCACCAGCAAGCT
**CD3e**	CTGCTACACACCAGCCTCAAA	TGGCTACTGCTGTCAGGTCC
**CD4**	TTTGCTGGTTCTGGCAACCT	ATCTTGGGAGAGGTAGGTCCC
**CD45**	CTCGCACCACTGAATCCACA	GATTGTCAGCTTGGCTGCTG
**CD68**	ACAAGGGACACTTCGGGCCA	GTCGTCTGCGGGTGATGCAG

Supplemental table 1: List of primer used in RT-PCR experiments to detect the listed mouse genes.

Sup Table 2: Physiological parameters

	Sham (5-7)	Sham HFD (7-10)
**Plasma Creatinine (µM)**	3.59±0.88	6.66±2.33
**Plasma Urea (mM)**	12.2±0.94	13.43±1.88
**Albuminuria (ng/24h)**	14918±718.4	14256±1608
**Body weight (gr/cm)**	15.2±0.78	21.98±0.82*
**Fat (%)**	11.67±1.62	20.87±2.03*
**EVAT (gr/cm)**	0.31±0.08	1.39±0.0.15*
**HbA1c (%)**	4.32±0.15	4.34±0.05

* $p<0.05$ vs Sham

Supplemental table 2: Physiological parameters of the groups Sham and Sham HFD measured at the sacrifice. The urine for albuminuria measurements was retrieved one week before sacrifice.
